# What defines hypervirulent *Klebsiella pneumoniae*?

**DOI:** 10.1016/j.ebiom.2024.105331

**Published:** 2024-09-10

**Authors:** Thomas D. Stanton, Kelly L. Wyres

**Affiliations:** a Department of Infectious Diseases, School of Translational Medicine, Monash University, Melbourne, Victoria 3004, Australia; b Centre to Impact AMR, Monash University, Clayton, Victoria 3800, Australia

*Klebsiella pneumoniae* is a global pathogen with remarkable genetic, phenotypic and pathogenic diversity.^1^ Strains belonging to distinct groups of lineages cause ‘classical’ infections in hospitals, often with high rates of multidrug resistance, or drug-susceptible ‘hypervirulent’ infections in community settings, respectively; however there are increasing reports of convergence between these pathotypes and their genetic determinants, raising significant public health concerns.^1^, ^2^, ^3^, ^4^ Sequence-type (ST) 23 strains are the dominant lineage causing hypervirulent infections, and associated with the K1 capsule type. Other common hypervirulent lineages are ST65, ST86 and ST66, associated with the K2 capsule. These strains are typically hypermucoviscous due to expression of the *rmpADC* hypermucoidy operon,^5^ and have enhanced iron acquisition via the production of siderophores (aerobactin, encoded by *iuc*; salmochelin, *iro*; yersiniabactin, *ybt*). The gene clusters encoding these features are usually carried on integrative-conjugative elements (*ybt*), or large plasmids (*iuc, iro, rmpADC* and its ortholog *rmpA2D2C2*). Several distinct so-called ‘virulence’ plasmids have been defined, but KpVP-1 is the most common and highly conserved among ST23, ST86 and ST65 strains. In contrast, KpVP-2 is conserved among ST66.^6^

There is evidence that hypermucoidy and siderophore production play roles in the hypervirulent phenotype,^7^ but experimental studies have typically focused on only a single strain, resulting in variable outcomes and a lack of clarity about the relative importance of these and other factors. In the recent issue of *eBioMedicine*,^8^ Russo and colleagues addressed this knowledge gap using an exemplary systematic analysis of four strains from hypervirulent infections, each harbouring KpVP-1 and representing a distinct lineage (common lineages ST23 and ST86, plus rarer ST29 and ST1544). Isogenic combinatory knockouts of KpVP-1, or *rmpA* and/or *iucA* and/or *irp2* (within the yersiniabactin operon) were generated as well as knockouts of *rmpA2* and *clbBC* (required for production of the colibactin genotoxin, present only in the ST23 strain). Each mutant was assessed for total siderophore production, mucoviscosity and lethal dose in a murine infection model - the gold-standard experimental approach for distinguishing hypervirulent from classical strains.^3^ The *iro* locus was not explored because the authors had previously shown that *iuc* results in the majority of extracellular siderophore production and has a greater impact on virulence in the ST86 strain.^9^

Loss of *rmpA* resulted in loss of hypermucoidy and the greatest single gene impact on lethal dose (mean 16.7-fold lethality decrease), followed by *iucA* (mean 9.6-fold decrease) and *irp2* (1.7-fold decrease). The impact of *rmpA* knockout was replicated for all four strains, but the impact of *iucA* knockout varied, with a significant LD_50_ decrease for only two strains; the ST86 strain that was used in the original comparative study of *iuc* and *iro*^9^*,* and the ST29 strain, but not ST23 nor ST1544. This led the authors to test three additional *iucA* mutants, a second ST29 strain, an ST893 and an ST375 (close relative of the common hypervirulent lineage, ST65). In all cases the loss of *iucA* had a significant impact on lethal dose.

Interestingly, combination *rmpA* and *iuc* knockouts resulted in a mean 20-fold lethality decrease that was far lesser than the impact observed when KpVP-1 was cured (67-fold decrease, significant for all four strains) and paled in comparison to the loss of KpVP-1 plus *irp2* (250-fold decrease). It is therefore clear that additional determinants located on KpVP-1 play a key role in hypervirulence, and that these interact with the *ybt* locus. Candidate plasmid-born factors include *peg-344*^10^ and those involved with iron sequestration e.g. *iro* and the ferric citrate operon (*fec*). Importantly, only a subset of these factors are encoded by KpVP-2 and the other known virulence plasmids,^6^ which may drive differential virulence impact. Together these data highlight the complexity of the hypervirulence phenotype and the importance of comparative analyses spanning multiple diverse strains, and multiple virulence plasmids.

Russo and colleagues concluded that KpVP-1 is the primary determinant of hypervirulence and primary target for novel therapeutics because only mutants lacking KpVP-1 were associated with LD_50_ values comparable to classical *K. pneumoniae*. We agree that the plasmid represents a key therapeutic target, but note that the current data do not demonstrate that KpVP-1 alone is sufficient to confer hypervirulence. Several studies have reported multidrug resistant classical *K. pneumoniae* which had acquired virulence plasmids,^3^^,^^4^^,^^8^ and only a subset of these were hypervirulent in mouse models. Strains that did not express hypervirulence spanned diverse STs and included those harbouring near complete copies of KpVP-1 with *rmpADC*, *iuc, iro* and *peg-344*.^4^^,^^8^ The key factors differentiating these strains are not known, but may include specific capsule types and other interacting determinants. A better understanding of these factors will inform accurate prediction of hypervirulence from genome data to aid global genomic surveillance efforts targeting hypervirulent and multidrug resistant *K. pneumoniae*.

## Contributors

KLW and TDS performed literature search and synthesis, drafted and edited the manuscript. Both authors read and approved the final manuscript.

## Declaration of interests

KLW is recipient of funding awards from the Bill and Melinda Gates Foundation to support development of genomic surveillance tools targeting *K. pneumoniae*.
